# Light Exposure Rhythms and Sleep Organization in Adolescents: Temporal Differences Between Weekdays and Weekends in an Actigraphic Study

**DOI:** 10.3390/clockssleep8020019

**Published:** 2026-04-15

**Authors:** Emilly Francianne Lamego da Silva, Guilherme Martins, Francimara Diniz Ribeiro, Leonardo Martins Guimaraes Rossi, Milena Fernandes de Oliveira, Camila Fernanda Cunha Brandão, Lucas Rios Drummond, Lucas Tulio Lacerda, Thais de Fatima Bittencourt Oliveira, Michael Jackson Oliveira de Andrade

**Affiliations:** 1 Laboratory of Neuroscience, Chronobiology, and Sleep Psychology, Room 101, State University of Minas Gerais, Divinopolis 35501-170, Brazil; 2 Laboratory for Research in Metabolism, Physiology, Physical Exercise, State University of Minas Gerais, Divinópolis 35501-170, Brazil; 3 Study and Research Group on Strength Training in Health, Physical Conditioning, State University of Minas Gerais, Divinópolis 35501-170, Brazil; 4 Research Group on Exercise, Sports Physiology, State University of Minas Gerais, Divinópolis 35501-170, Brazil

**Keywords:** adolescents, light exposure, circadian rhythms

## Abstract

Light exposure is a primary zeitgeber for the human circadian system and plays a key role in shaping sleep–wake patterns during adolescence, a period marked by biological sensitivity and social constraints. How the temporal organization and spectral composition of daily light exposure differ between weekdays and weekends remains poorly understood. Eighteen adolescents (15–17 years) were monitored for seven days using wrist actigraphy with integrated light sensors. Sleep parameters, nonparametric circadian rhythm indices, and time-resolved profiles of ambient and spectral (blue, green, and red) light exposure were analyzed. Repeated-measures ANOVA tested the effects of time of day and day type. Total sleep time and time in bed were longer on weekdays than on weekends (*p* < 0.05), while sleep latency and WASO did not differ. Circadian indices indicated preserved rhythmic organization. Light exposure showed a robust diurnal profile, with higher spectral irradiance on weekends (*p* < 0.001), especially in the morning and early afternoon. Significant time × day-type interactions were observed across all spectral bands (*p* < 0.001), indicating systematic reshaping of daily light profiles. Adolescents exhibit weekday–weekend differences in the temporal and spectral organization of light exposure, affecting the amplitude and shape of overall daily profiles.

## 1. Introduction

The ambient light plays a central role in the temporal organization of human biological rhythms, influencing not only visual processes but also a wide range of non-visual responses, including the regulation of the sleep–wake cycle, melatonin secretion, and levels of alertness [1,2]. Light detection and processing by the retina involve multiple classes of photoreceptors, including rods and cones, which mediate classical visual functions, as well as intrinsically photosensitive retinal ganglion cells (ipRGCs), which play a fundamental role in circadian synchronization [3,4].

The ipRGCs express melanopsin and exhibit greater spectral sensitivity to short wavelengths, particularly in the range of 460–490 nm, constituting the primary pathway for transmitting photic information to the suprachiasmatic nucleus (SCN) of the hypothalamus, the main circadian pacemaker in mammals [2,3]. Activation of these pathways is directly associated with melatonin suppression and the modulation of wakefulness, making light exposure a critical determinant of sleep quality and regularity, traditionally recognized as the principal photic zeitgeber of the circadian system [5,6].

Recent advances in the chronobiology of light have emphasized the need to characterize light exposure using standardized metrics that account for the spectral sensitivity of different retinal photopigments. In this context, the International Commission on Illumination (CIE) proposed the CIE S 026:2018 standard, which defines α-opic spectral weighting functions and metrics such as melanopic equivalent daylight illuminance (melanopic EDI), now widely recommended for the assessment of non-visual circadian responses to light [7]. Complementarily, the guidelines of the Expert Network on Light Intervention and Neurophysiology (ENLIGHT) provide specific recommendations for the reporting and interpretation of light exposure in human field studies [7,8].

During adolescence, maturational changes in the circadian and homeostatic sleep systems interact with social and academic demands that frequently impose early wake times and irregular patterns of light exposure. In the Brazilian school context, morning classes typically begin between 7:00 and 7:30 a.m. and end around 12:00 or 12:30 p.m., whereas afternoon classes usually run from approximately 1:00 p.m. to 6:00 p.m. In full-time school schedules, the school day often starts at 7:00 a.m. and extends until 4:00 or even 6:00 p.m., intensifying early wake times and prolonged periods of continuous wakefulness. This scenario may exacerbate the misalignment between adolescents’ biological rhythms and social demands, particularly when combined with low daytime light exposure and residual exposure to light at night [8,9,10]. Observational evidence indicates that patterns characterized by reduced daytime light exposure and residual nocturnal light exposure are associated with shorter sleep duration, longer sleep onset latency, and poorer sleep efficiency in this age group [9,10].

Moreover, systematic differences between weekdays and weekends have been widely described as a marker of circadian misalignment, often referred to as social jetlag, reflecting shifts in sleep timing, wake times, and ambient light exposure [4]. Biological factors, such as sex, may further modulate light sensitivity and sleep organization during adolescence, due to hormonal differences and pubertal stage [10].

Although the literature has consistently demonstrated that light exposure influences sleep and circadian regulation in adolescents, most studies have focused on global measures of light intensity or on restricted time windows, often without considering the temporal organization of exposure across the 24 h cycle. In addition, relatively few investigations have systematically contrasted weekdays and weekends, despite these contexts representing distinct social and behavioral time regimes during adolescence.

In light of these gaps, the present study aimed to characterize diurnal profiles of ambient and spectral light exposure, interpreted as relative indicators of integrated irradiance, and to examine their relationship with sleep parameters, explicitly contrasting weekdays and weekends. By emphasizing the temporal organization of light exposure, this study seeks to provide a more ecologically valid understanding of how social time modulates light environments that are relevant to circadian health in adolescents.

## 2. Results

### 2.1. Sleep Parameters and Behavioral Context

All participants reported using electronic devices throughout the day, including smartphones, laptops, and televisions. Eighty-six percent (86%) reported having a television and/or computer in the bedroom, indicating broad availability of these devices in the sleep environment. In addition, 100% of participants reported smartphone use close to sleep periods, including before bedtime, during nighttime awakenings, and upon waking in the morning. Based on self-report, total daily screen time showed substantial inter-individual variability, averaging approximately 2 to 3 h per day. All participants also reported using electronic alarms to wake up on weekdays. Regarding subjective sleep quality, approximately 22% rated their sleep as fair or poor, whereas about 78% rated it as good or very good.

As shown in Table 1, significant differences between weekdays (DS) and weekends (FDS) were observed in sleep duration parameters. Time in bed was significantly longer on weekdays than on weekends (t(30) = 2.10, *p* = 0.045), with a moderate effect size (d = 0.74). Similarly, total sleep time was significantly greater on weekdays (t(30) = 2.55, *p* = 0.016), corresponding to a moderate-to-large effect (d = 0.90). In contrast, sleep onset latency (*p* = 0.347) and wake after sleep onset (WASO) (*p* = 0.743) did not differ significantly between day types.

Complementarily, the nonparametric indices, calculated based on weekdays, indicated preservation of basal circadian organization. Interdaily stability (IS) showed a mean value of 0.44 (SD = 0.11; 95% CI = 0.43–0.46), reflecting moderate regularity of the daily pattern across days. Intradaily variability (IV) presented a mean of 0.69 (SD = 0.18; 95% CI = 0.68–0.71), indicating moderate intradaily fragmentation.

With respect to the temporal distribution of activity and rest, the most active 10 h block (M10) exhibited high mean values (M = 3687.24; SD = 1137.26), with an average onset at 08:22 h, indicating consolidation of daytime wakefulness under strong social synchronization. Conversely, the least active 5 h block (L5) showed low mean values (M = 434.19; SD = 242.65), with an average onset at 04:04 h, reflecting relatively consolidated nocturnal rest.

Relative amplitude (RA) was high (M = 0.86; SD = 0.05; 95% CI = 0.82–0.90), indicating a strong contrast between daytime activity and nighttime rest. Taken together, these findings suggest that, despite quantitative differences in sleep duration between weekdays and weekends, the sleep–wake rhythm remained relatively stable, organized, and functionally differentiated in a context of strong social synchronization.

### 2.2. Temperature

External temperature and wrist skin temperature exhibited systematic variation across the 24 h cycle, with observable differences between weekdays and weekends.

Regarding external temperature, a significant main effect of time of day was observed (F(11, 11,045) = 733.04; *p* < 0.001; η^2^p = 0.422), indicating systematic variation across the 24 h cycle. A significant interaction between time of day and day type (weekdays vs. weekends) was also detected (F(11, 11,045) = 123.27; *p* < 0.001; η^2^p = 0.109), suggesting differences in the daily profile of external temperature between weekdays and weekends, systematically between weekly contexts. In both conditions, temperature peaks occurred in the late evening (22:00–24:00), consistent with typical daily variation in environmental temperature, whereas minimum values were observed in the early morning hours (06:00–10:00).

Overall, mean external temperature was slightly but significantly higher on weekends compared to weekdays (mean difference = 0.13 °C; F(1, 11,055) = 27.18; *p* < 0.001; η^2^p = 0.002), with a small effect size. Wrist skin temperature reflects peripheral skin temperature measured at the wrist and may be influenced by environmental conditions and behavioral factors [11].

### 2.3. Ambient Light Irradiance

**Figure 1:**
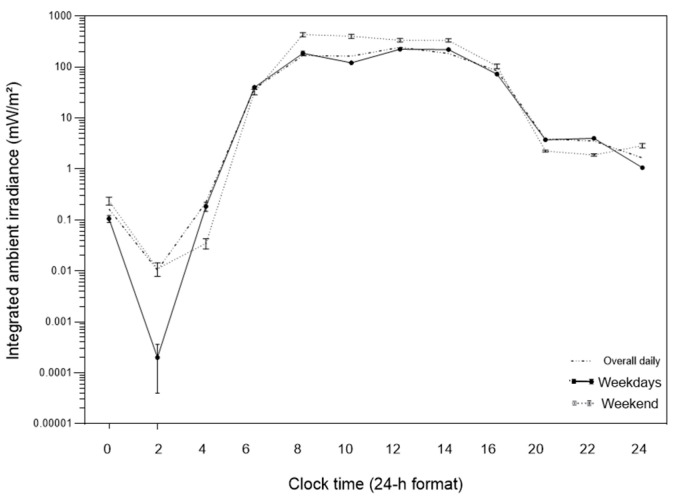
Twenty-four-hour profile of ambient light exposure expressed as integrated irradiance (mW/m^2^) across the daily cycle. Solid lines represent weekdays (DS), dashed lines represent weekends (FDS), and the dotted line represents the overall mean daily profile. Values are displayed on a logarithmic scale and presented as mean ± standard error of the mean (SEM), aggregated in 2 h time bins to characterize daily variation in environmental light exposure.

The mixed-design analysis of variance revealed a clear variation across the 24 h cycle of ambient light exposure across the 24 h cycle, as well as significant differences as a function of day type (Figure 1). Ambient light exposure exhibited a well-defined diurnal profile, with minimal values during the late night and early morning hours (00:00–06:00) and peak values concentrated between 08:00 and 16:00, characterizing a high-amplitude daily pattern.

A significant main effect of time was observed (V = 0.422; F(11, 5506) = 365.71; *p* < 0.001; η^2^p = 0.422), indicating marked variation across the 12 two-hour time windows. A significant main effect of day type was also detected (F(1, 5516) = 140.10; *p* < 0.001; η^2^p = 0.025), with higher mean ambient light exposure on weekends (138.15 ± 4.67) compared to weekdays (72.90 ± 2.93).

Importantly, these global differences were time-of-day dependent. In the early morning (06:00–08:00), exposure was slightly higher on weekdays (DS = 39.99; FDS = 32.71). In contrast, during the 08:00–10:00 interval, a marked inversion was observed, with substantially higher exposure on weekends (DS = 186.20; FDS = 435.27), corresponding to an absolute difference of +249.06 units and a FDS/DS ratio of 2.34, representing the main window of temporal discrepancy between day types (*p* < 0.05).

In the early evening (18:00–20:00), exposure again became higher on weekdays (DS = 3.76; FDS = 2.25; FDS/DS ratio = 0.60), indicating greater persistence of light exposure after sunset on school days.

### 2.4. Short-Wavelength Lighting Irradiance (BL)

Short-wavelength lighting exposure was virtually absent during the late night and early morning hours (00:00–06:00) in both day-types. A significant main effect of time and a significant time × day type interaction were observed (*p* < 0.05). Post hoc analyses indicated that exposure increased from the morning onward (06:00–08:00) in both conditions, with a significantly steeper rise on weekends compared to weekdays. Peak exposure occurred between 08:00 and 16:00, with significantly higher levels on weekends, particularly in the 08:00–10:00, 10:00–12:00, and 14:00–16:00 intervals. During the late afternoon and night (18:00–24:00), exposure returned to low levels in both conditions, although some time-specific differences remained (Table 2).

Multivariate analyses revealed a strong main effect of time (V = 0.399; F(11, 4939) = 297.58; *p* < 0.001; η^2^p = 0.399), as well as a significant time × day-type interaction (V = 0.066; F(11, 4939) = 31.61; *p* < 0.001; η^2^p = 0.066). Mauchly’s test indicated violation of sphericity (*p* < 0.001), and the Greenhouse–Geisser correction was therefore applied (ε = 0.319). Under this correction, statistically significant effects of time (F(3.50, 17,341.74) = 185; η^2^p = 0.036) and the time × day-type interaction (F(3.50, 17,341.74) = 22.2; η^2^p = 0.004) were maintained.

Polynomial contrasts indicated that temporal variation in short-wavelength lighting exposure was predominantly explained by nonlinear components, with a robust quadratic effect (F(1, 4949) = 1165.86; *p* < 0.001; η^2^p = 0.191), as well as significant higher-order components, evidencing a complex temporal structure across the 24 h cycle.

### 2.5. Middle-Wavelength Light Irradiance (GL)

Middle-wavelength light exposure exhibited a well-defined diurnal temporal profile, with minimal values during the late night, a progressive increase throughout the morning, and a marked reduction during the night. The largest differences between weekdays and weekends were concentrated in the morning period (06:00–12:00), with substantially higher exposure on weekends, particularly after 08:00. During the afternoon (12:00–18:00), exposure remained elevated in both contexts, but was consistently higher on weekends. At night (18:00–24:00), levels returned to residual values, with small-magnitude differences (Table 2).

Multivariate tests revealed a robust main effect of time (V = 0.434; F(11, 5467) = 380.69; *p* < 0.001; η^2^p = 0.434), as well as a significant time × day-type interaction (V = 0.078; F(11, 5467) = 41.84; *p* < 0.001; η^2^p = 0.078). Given the violation of sphericity (*p* < 0.001), univariate tests were interpreted using the Greenhouse–Geisser correction (ε = 0.347), which preserved the significance of both the main effect of time (F(3.81, 20,887.02) = 244.26; *p* < 0.001; η^2^p = 0.043) and the time × day-type interaction (F(3.81, 20,887.02) = 36.19; *p* < 0.001; η^2^p = 0.007).

At the between-subjects level, mean exposure was higher on weekends (FDS = 68.88 vs. DS = 36.21; Δ = 32.66; *p* < 0.001). Polynomial contrasts indicated the predominance of nonlinear components, with a strong contribution of the quadratic term (η^2^p = 0.223), suggesting differences in both the shape and amplitude of the diurnal profile between day types. Parameter estimates by time interval indicated that differences between weekdays and weekends were predominantly concentrated during the daytime, especially between 08:00 and 14:00, with higher middle-wavelength light exposure on weekends, whereas nighttime differences were of smaller magnitude and variable direction.

### 2.6. Long-Wavelength Light Irradiance (RL)

Long-wavelength light exposure exhibited a well-defined diurnal temporal profile, with near-zero values during the late night and early morning (00:00–06:00), followed by a sharp increase from the morning onward, reaching peak levels between 08:00 and 16:00. Multivariate tests confirmed a robust main effect of time (V = 0.407; F(11, 5349) = 334.31; *p* < 0.001; η^2^p = 0.407), as well as a significant time × day-type interaction (V = 0.069; F(11, 5349) = 35.82; *p* < 0.001; η^2^p = 0.069).

Violation of sphericity (*p* < 0.001) motivated the use of the Greenhouse–Geisser correction (ε = 0.325), under which significant effects of time (F(3.58, 19,166.05) = 184.95; *p* < 0.001; η^2^p = 0.033) and the time × day-type interaction (F(3.58, 19,166.05) = 23.43; *p* < 0.001; η^2^p = 0.004) were preserved. At the between-subjects level, mean long-wavelength light exposure was higher on weekends (DS = 23.61 vs. FDS = 42.88; Δ = 19.27; F(1, 5359) = 104.11; *p* < 0.001; η^2^p = 0.019).

Polynomial contrasts indicated a predominance of nonlinear components, with marked contributions of the quadratic (η^2^p = 0.181) and fourth-order (η^2^p = 0.153) terms, suggesting differences in both the shape and amplitude of the diurnal profile between weekdays and weekends. Descriptively, discrepancies were concentrated during the daytime, particularly between 08:00 and 12:00, whereas during the night (18:00–24:00) exposure returned to residual levels in both day types (Table 2).

## 3. Discussion

### 3.1. Spectral Profile and Temporal Organization of Daily Light Exposure

Parameter estimates by time interval consistently showed that differences between weekdays and weekends in blue, green, and long-wavelength light exposure were strongly time-of-day dependent, concentrating predominantly during the daytime, especially between 08:00 and 14:00. This pattern is consistent with classical models of human chronobiology, according to which the circadian system is primarily entrained by the environmental light–dark contrast, but its daily expression is strongly modulated by social time and behavioral routines [3,12,13]. Contemporary clinical guidelines and reviews further emphasize that everyday light exposure should be understood as an active environmental determinant of sleep–wake temporal organization, with direct implications for the risk of functional circadian misalignment, particularly in contexts of high social constraint [14,15].

The higher light exposure observed on weekends suggests that social time primarily modulates the amplitude and shape of the daily exposure profile, without implying changes in circadian phase. This finding converges with ambulatory studies showing that adolescents and young adults exhibit lower exposure to natural daylight on weekdays and higher daytime exposure on free days, reflecting restrictions imposed by school and academic routines [16]. Thus, reduced effective daytime light exposure in enclosed school environments, when combined with residual light exposure in the early evening, may diminish the functional contrast between day and night, increasing vulnerability to phase delay in adolescents, even in the absence of detectable structural circadian disorganization [17,18,19].

In this context, schools should be recognized as central modulators of daytime light exposure, especially during the morning, which represents the time window of greatest chronobiological efficacy of light. Classrooms with ample access to natural daylight favor greater exposure to short- and medium-wavelength light, particularly in the blue range (460–500 nm), which is associated with stronger melanopic activation and enhanced morning circadian entrainment [5,12].

Importantly, however, despite the observed differences in morning and daytime light exposure between weekdays and weekends, no measurable alterations in basal circadian rhythmicity were detected in the present sample. Nonparametric indices and rest–activity organization remained relatively stable, indicating that the reduced weekday morning light exposure in school settings was not associated with detectable circadian disruption under the ecological conditions evaluated. Thus, the present findings should be interpreted as reflecting modulation of exposure profiles rather than demonstrable circadian consequences.

An additional methodological consideration should be emphasized when interpreting the circadian and non-visual implications of the present findings. Light exposure in this study was characterized using relative spectral irradiance rather than standardized α-opic metrics, such as melanopic equivalent daylight illuminance (melanopic EDI), as recommended by the CIE S 026:2018 and ENLIGHT guidelines. Therefore, all inferences regarding the potential “non-visual” circadian impact of light should be interpreted as indirect and based on the temporal and spectral organization of exposure rather than on direct quantification of effective melanopic stimulation.

Although the spectral distribution in the short-wavelength range suggests potential circadian relevance, the absence of calibrated α-opic metrics precludes precise estimation of melanopic dose and ipRGC-weighted stimulation. Future studies should incorporate spectrally calibrated sensors and standardized α-opic measures (e.g., melanopic EDI, melanopic irradiance) to allow more accurate and physiologically grounded assessment of circadian-effective light exposure in real-world adolescent settings.

### 3.2. Rest–Activity Rhythm, Circadian Stability, and Intradaily Variability

Despite quantitative differences in sleep duration between weekdays and weekends, nonparametric indices indicated preservation of basal circadian organization. This preservation occurred despite the documented differences in light exposure profiles, reinforcing the interpretation that environmental variations in ecological light exposure were insufficient to induce detectable circadian instability in this cohort. Moderate values of interdaily stability and intradaily variability, combined with relatively high amplitude, are compatible with a functionally differentiated sleep–wake rhythm, characterized by consolidated daytime wakefulness (high M10) and relatively preserved nocturnal rest (low L5). This pattern is consistent with normative descriptions of rest–activity rhythms in adolescent populations under strong social synchronization, in which intradaily fragmentation predominantly reflects intra-day behavioral heterogeneity rather than primary instability of the central circadian oscillator [4,13].

From a clinical perspective, international guidelines emphasize that quantitative changes in sleep, when not accompanied by a loss of rhythmic stability, should be interpreted as temporal and behavioral adjustments rather than as circadian sleep–wake rhythm disorders [14].

The methodological decision to compute nonparametric indices based exclusively on weekdays is supported by these recommendations, as such metrics rely on multiple consecutive days for reliable estimation. Very short time windows, such as isolated weekends, tend to inflate variance and reduce interpretative validity, particularly in adolescents, whose behavior shows high contextual variability [4]. In this sense, the relative preservation of nonparametric parameters observed in this study suggests that the identified pattern reflects a functional adaptation to predictable social constraints rather than a primary circadian disorganization.

### 3.3. Evening and Nighttime Light Exposure, Circadian Sensitivity, and Phase Delay

Although the largest spectral differences occurred during the daytime, the pattern observed in the early evening (approximately between 18:00 and 22:00) has chronobiological relevance disproportionate to its absolute magnitude. Experimental evidence shows that this interval corresponds to a phase of heightened circadian sensitivity to light, as described by phase response curves, in which nocturnal light exposure produces robust phase delays.

Experimental and observational studies indicate that even moderate light exposure, when combining sufficient duration and short-wavelength or broadband spectral components, can suppress melatonin secretion and delay sleep onset, with particularly pronounced effects in adolescents [17,20,21,22].

In this context, accumulating evidence suggests that adolescence represents a specific window of circadian vulnerability, in which the physiological delay of the biological clock interacts with increased sensitivity to evening and nighttime light. Recent reviews indicate that exposure to artificial light at night is associated not only with sleep disturbances but also with changes in alertness, cognitive functioning, and emotional regulation, particularly in young populations exposed to intense social demands—such as those reflected in the self-reported electronic device use in the present sample [15,18].

Notably, in contrast to the commonly assumed association between evening light exposure and sleep restriction, adolescents in the present study slept longer on weekdays than on weekends. This finding suggests that strong social synchronization, particularly structured school schedules, may have overridden potential delaying effects of evening light exposure on sleep timing.

Therefore, although early evening light exposure has been recognized as having chronobiological relevance according to phase response curves, the present data do not provide direct evidence of cumulative phase delays or circadian disruption. Instead, the observed pattern is more consistent with behavioral and social regulation of sleep–wake schedules rather than light-driven circadian misalignment under the ecological conditions assessed.

### 3.4. Spectral Composition, Social Time, and Implications for Adolescent Sleep Health

Although short-wavelength lighting is recognized as the primary chronobiologically active stimulus, the present results show that the temporal profiles of green and long-wavelength light closely track the pattern of short-wavelength light throughout the day. This finding reinforces the notion that, in real-world ecological environments, light exposure occurs as a composite spectral stimulus rather than as isolated wavelengths.

Recent evidence indicates that circadian responses to light depend on the interaction between intensity, spectrum, and duration of exposure, with nonlinear temporal dynamics [17].

From a social time perspective, these findings directly engage with the literature linking school organization and adolescent sleep health. Systematic reviews have shown that early school start times are associated with shorter sleep duration, poorer sustained attention, and greater daytime sleepiness, even when circadian rhythms remain structurally organized [14,18].

The coexistence of reduced effective daytime light exposure on weekdays, increased light exposure on weekends, and frequent nighttime use of light-emitting devices creates an environment conducive to misalignment between biological time and social time. Accordingly, the present data support the need for integrated approaches that simultaneously consider school schedules, daytime light exposure, and nighttime light control, particularly in full-time schooling contexts.

Importantly, the relatively homogeneous spectral distribution of red, green, and short-wavelength lighting observed across daily conditions suggests that ecological circadian light exposure in adolescents is predominantly broadband rather than dominated by isolated wavelengths. This has practical implications for circadian light mitigation strategies, such as device-based “circadian-friendly” or blue-light–reduction modes. While spectral filtering may contribute to reducing short-wavelength exposure, the present findings indicate that timing, duration, and overall light exposure patterns may play a more relevant role than isolated spectral composition alone in shaping real-world circadian and sleep behavior.

### 3.5. Ambient Temperature, Thermoregulation, and Sleep–Wake Transitions

In addition to light exposure, the ambient temperature and wrist skin temperature profiles observed in this study provide a relevant physiological context for sleep–wake organization. The presence of systematic daily variation in temperature, with higher values in the late night and minima in the early morning, is consistent with the literature describing the close interaction between thermoregulation and sleep.

Previous physiological studies show that sleep onset generally occurs during the descending phase of core body temperature, accompanied by increased peripheral vasodilation and elevation of distal skin temperature, whereas awakening is associated with the opposite process [11,23].

Actigraphy-based studies with integrated thermal sensors indicate that wrist skin temperature tends to increase around sleep onset and decrease after awakening, representing a useful ecological marker of sleep–wake transitions under real-world conditions [24].

In this context, the small but systematic differences between weekdays and weekends in ambient temperature profiles observed in this study may represent contextual environmental factors that co-vary with sleep timing, interacting with light exposure and social schedules without necessarily producing detectable changes in global circadian stability.

Alternatively, the slightly higher nocturnal thermal nadir observed on weekends may reflect behavioral masking effects, such as increased physical activity, later bedtimes, or greater environmental and social engagement during free days, rather than intrinsic circadian mechanisms. This interpretation is consistent with ecological actigraphy studies indicating that peripheral temperature patterns can be modulated by behavioral context independently of central circadian phase.

Together, these findings reinforce the importance of jointly considering light exposure, environmental temperature, and behavioral context as interacting environmental cues influencing adolescent sleep under real-world conditions.

### 3.6. Conceptual Interpretation Based on Circadian Principles

The findings of the present study may be conceptually interpreted in relation to classical circadian principles, rather than as direct evidence of circadian regulation. Although no circadian phase markers were assessed, the observed patterns of light exposure and sleep–wake organization may reflect behavioral and environmental dynamics that are broadly consistent with circadian organization. In this context, references to circadian concepts, including those historically described by Pittendrigh, are intended as a theoretical framework to contextualize the findings, rather than as empirical support for endogenous circadian mechanisms or precise temporal regulation [19,21,22].

Within this framework, the absence of global shifts in the sleep–wake rhythm between weekdays and weekends, concomitant with modulation of the shape and amplitude of light exposure profiles, suggests preservation of central oscillator organization, with variations occurring primarily in the daily expression of the rhythm.

This interpretation is reinforced by the relative stability of nonparametric indices, consistent with the notion that biological clocks maintain temporal regularity even under predictable environmental fluctuations. The observed patterns are therefore more consistent with functional adjustments mediated by social synchronizers than with primary circadian instability. More specifically, the preservation of interdaily stability (IS) and intradaily variability (IV) indices reinforces the interpretation of a robust circadian organization despite environmental fluctuations in light exposure. According to Pittendrigh’s generalizations, circadian systems exhibit intrinsic stability and resistance to perturbations, maintaining temporal coherence even when exposed to moderate variations in external zeitgebers. In this context, the stability of IS and IV suggests that the central circadian pacemaker remained functionally synchronized, while environmental light differences primarily affected the amplitude and temporal distribution of daily activity patterns rather than the underlying circadian structure.

The results also support the idea that circadian clocks are open systems whose phase is modulated by environmental synchronizers, with light playing a central role. The concentration of light exposure differences within windows of higher functional relevance—especially in the early evening—is compatible with the concept of phase response curves, according to which the chronobiological impact of light critically depends on the timing of exposure.

Thus, modest variations in evening and nighttime light exposure may influence sleep timing and daily behavioral organization; however, no direct evidence of cumulative phase delay was observed in the present study, and interpretations regarding phase dynamics should be considered cautiously.

Complementarily, the observed thermal profiles suggest that temperature acts as a contextual modulator of sleep–wake transitions, influencing thermal comfort and heat dissipation without inducing detectable changes in basal circadian stability [11,23]. This preservation of temporal organization despite environmental fluctuations is consistent with Pittendrigh’s principle of compensation and robustness, reinforcing the view that light and temperature should be interpreted as integrated environmental signals.

Finally, in line with Pittendrigh’s framework, the identified patterns suggest a regime of functional adaptation to predictable social constraints rather than primary circadian disorganization. This perspective shifts interpretation from a rhythm disorder model to a cumulative temporal adjustment model, with direct implications for environmental interventions in school contexts.

### 3.7. Limitations and Future Directions

Despite the robustness of the temporal and spectral analyses, this study has limitations that should be considered when interpreting the findings. First, light exposure was characterized using broad spectral bands, which did not allow direct estimation of standardized α-opic metrics, such as melanopic equivalent daylight illuminance (melanopic EDI), as recommended by the CIE S 026:2018 and ENLIGHT guidelines [7].

Accordingly, inferences about circadian impact should be understood as indirect and based on the temporal and spectral organization of exposure rather than on direct measures of effective melanopic dose—a limitation widely recognized in field studies using actigraphy and portable light sensors [13,25]. Nevertheless, approaches based on temporal exposure profiles have been considered valid for ecological inferences about circadian entrainment and sleep–wake behavior in young populations [26].

Additionally, the observational design of the study precludes causal inferences between light exposure and sleep outcomes, although the observed patterns are consistent with the existing chronobiological and clinical literature demonstrating time-, duration-, and spectrum-dependent effects of light on the human circadian system [14,15,20].

Experimental and clinical evidence indicates that light acts both through circadian mechanisms (via modulation of phase and rhythmic amplitude) and through direct effects on alertness, attention, and homeostatic sleep pressure, reinforcing the need for caution in causal interpretation of observational findings [3].

A specific methodological point concerns the nonparametric indices (IV, IS, L5, M10, and RA), which were calculated exclusively based on weekdays. This decision is supported by the fact that these metrics rely on stable estimates of regularity and fragmentation across multiple consecutive days. Methodological guidelines and validation studies in actigraphy indicate that nonparametric indices show greater stability and validity when derived from longer time series under relatively consistent routines [27].

An additional limitation relates to the sample characteristics. The study was conducted with a relatively small sample (*n*N = 18) recruited from a single geographic region and from a specific educational context (full-time school system) in Brazil, which limits the generalizability of the findings. Adolescents enrolled in full-time schooling are exposed to more structured daily schedules and stronger social synchronizers, which may influence sleep–wake patterns, light exposure routines, and circadian organization. Therefore, the observed preservation of basal circadian stability indices (e.g., IS and IV) and the identified light exposure profiles should be interpreted with caution, as they may reflect context-dependent environmental and institutional constraints rather than patterns representative of the broader adolescent population. If feasible, future studies should include photoperiod length as a covariate in ANOVA models or directly compare weeks with different daylight durations to better control for seasonal light variability. In addition, future research should adopt higher temporal resolution (e.g., shorter time bins), smoothed curves, and time-series modeling approaches to more accurately capture the circadian dynamics of light exposure across the 24 h cycle.

Moreover, the regional recruitment and the specificity of the school model may not capture the variability present across different socioeconomic, cultural, and educational contexts. Future studies should include larger and multicenter samples, encompassing diverse regions, school schedules (full-time and part-time), and sociocultural backgrounds, to enhance external validity and allow more robust generalization of chronobiological findings in adolescents.

Thus, restricting these indices to weekdays—characterized by a greater number of observed days and stronger social synchronization—prioritizes internal validity and comparability of basal circadian organization parameters [10].

Finally, future studies should integrate calibrated spectral measurements, α-opic metrics, and direct physiological markers such as salivary melatonin or objective phase estimates, as recommended by recent reviews and clinical chronobiology guidelines [20,25].

Longitudinal designs and intervention studies exploring environmental modifications—including school lighting strategies, nighttime light-emitting device control, and adjustments to academic schedules—may further contribute to a more translational understanding of how light exposure modulation affects adolescent sleep health, particularly in full-time schooling contexts with high temporal pressure [9,26].

## 4. Materials and Methods

### 4.1. Participants

Eighteen adolescents aged 15 to 17 years participated in the study (10 girls and 8 boys). All were regularly enrolled in a public full-time high school (08:00 a.m. to 5:30 p.m.) and followed a similar school routine, with classes starting around 8:00 a.m. The study was approved by the Research Ethics Committee of the Universidade do Estado de Minas Gerais (CAAE: 69328623.8.0000.5115). Legal guardians provided written informed consent, and all adolescents gave written assent.

The sample size (*n* = 18) was determined by feasibility constraints related to ambulatory monitoring in a controlled school context and device availability. Considering the within-subject design (weekdays vs. weekends), which increases statistical efficiency by reducing inter-individual variability, this sample provides adequate power to detect moderate effects (α = 0.05) in paired comparisons. However, the study may be underpowered to detect small effects, which increases uncertainty and limits the generalizability of the findings. Accordingly, results should be interpreted with caution, and larger multicenter studies are recommended for more precise effect estimation.

### 4.2. Study

Data collection was conducted in the city of Divinópolis, Minas Gerais, Brazil (20° S, 44° W), a medium-sized urban area in Southeastern Brazil. The region presents moderate seasonal variation in photoperiod. The data collection occurred between May and July 2025, corresponding to the austral autumn–winter period, with an average daylight duration of approximately 8.1 h. This reduced photoperiod may have influenced the 24 h light exposure profiles, particularly by limiting natural light availability in the early morning and late afternoon hours on weekdays. Therefore, the observed daily light exposure patterns should be interpreted considering the seasonal photoperiod context, which may partially contribute to lower morning illuminance and prolonged evening exposure to artificial light. In addition, diurnal temperature variation was observed, with mean temperatures of approximately 16 °C in the morning and 26 °C in the afternoon.

During school days, adolescents spent most of their time indoors. Exposure to natural light occurred mainly during school breaks, lasting approximately 30 min in the morning and 30 min in the afternoon, as well as during brief daily commuting periods. Some classrooms lacked windows and consisted of enclosed environments with continuous artificial lighting and air-conditioning, substantially restricting the entry of natural daylight during academic activities.

### 4.3. Study Design and Procedure

This was an observational field study. Participants were continuously monitored using actigraphy for seven consecutive days (168 h), including five weekdays and two weekend days. This period was considered sufficient to capture typical variability between school routines and non-school days, in accordance with the recommendations of the Brazilian Consensus on Actigraphy [28].

The device was worn on the non-dominant wrist throughout the entire recording period, enabling continuous monitoring of activity–rest patterns and ambient light exposure across the 24 h cycle. Participants were instructed to maintain their habitual sleep–wake routines, with no experimental manipulation. Data collection occurred during a regular school term, outside of examination weeks or atypical school events.

### 4.4. Instruments

#### 4.4.1. Sociodemographic Questionnaire

A structured sociodemographic questionnaire was specifically developed for the present study to characterize the participants’ profiles. The instrument included items related to age, sex, school grade, school schedule, daily routine, use of electronic devices, physical activity, general sleep habits, and self-reported health conditions. These data were used for sample description and to support exploratory and statistical control analyses.

#### 4.4.2. ActTrust Actigraph

Objective sleep–wake parameters and ambient light exposure were recorded using a wrist-worn ActTrust^®^ Actigraph (Condor Instruments, São Paulo, Brazil; ActTrust 2 model). The device enables continuous ambulatory monitoring of motor activity, wrist skin temperature, and environmental light exposure under ecological conditions. Motor activity was recorded via a triaxial accelerometer, while light exposure was measured using an integrated multichannel spectral sensor (red, green, and blue channels) capable of estimating relative spectral irradiance and photopic illuminance.

Prior to deployment, all devices were time-synchronized and inspected according to the manufacturer’s recommendations. Factory calibration provided by the manufacturer was maintained, and sensor functionality (accelerometer, light, and temperature) and battery status were verified before each recording period to ensure data consistency across participants.

Data were sampled in 60 s epochs, allowing continuous 24 h monitoring, including sleep periods. Participants were instructed to wear the device continuously on the non-dominant wrist throughout the monitoring period and to remove it only when strictly necessary (e.g., activities that could damage the device). A brief familiarization and standardized instruction session was conducted before data collection to ensure compliance and minimize behavioral reactivity to device use.

The device includes an off-wrist detection sensor, which was used for compliance verification and data quality control. Periods of non-wear were identified based on off-wrist detection signals, sustained zero-activity intervals, and abrupt temperature drops, and were excluded from subsequent analyses.

Data extraction, preprocessing, and analysis were performed using ActStudio softwareversion 2.0 (Condor Instruments), following the manufacturer’s specifications and standardized actigraphy processing procedures. Raw data were visually inspected to identify artifacts and missing segments, and non-physiological patterns or confirmed non-wear periods were removed prior to parameter calculation.

#### 4.4.3. Outcome Measures

*Sleep and Circadian Parameters:* Actigraphic recordings were used to derive objective sleep parameters and nonparametric indices of circadian organization. Sleep variables included total sleep time, sleep onset latency, wake after sleep onset, number of awakenings, and sleep efficiency. Sleep–wake states were automatically scored using validated algorithms, including Actiware (Philips Respironics) and ActStudio (based on the Cole–Kripke algorithm). These algorithms classify sleep and wake periods based on activity patterns, from which standard sleep parameters were computed.

Nonparametric rest–activity rhythm parameters (interdaily stability [IS], intradaily variability [IV], least active 5 h [L5], and most active 10 h [M10]) were calculated exclusively based on weekdays. IS quantified the day-to-day regularity of activity patterns, whereas IV measured the fragmentation of the rest–activity rhythm within days. L5 and M10 were defined as the consecutive 5 h and 10 h periods with the lowest and highest mean activity levels, respectively, based on activity counts. This decision was made because these metrics require sufficiently long and stable time series to reliably estimate circadian regularity and fragmentation. Since the weekend period comprised only two consecutive days, their isolated inclusion would not meet the methodological assumptions of these measures and could introduce bias into the estimation of rhythmic indices [28].

*Temperature:* External temperature corresponds to ambient temperature recorded by the Actigraph devices, whereas wrist temperature reflects peripheral skin temperature measured by the integrated thermistor of the wrist-worn device [28].

*Light Exposure:* Light exposure was continuously recorded using a multichannel spectral sensor integrated into the Actigraph, allowing simultaneous collection of total ambient light exposure and relative irradiance in different spectral bands (red, green, and blue). Polychromatic light exposure with spectral peaks in the short-, middle-, and long-wavelength ranges (approximately 460–480 nm, 520–540 nm, and 620–660 nm, respectively) was analyzed. Total ambient light exposure was used as a global indicator of light intensity, whereas spectral bands were analyzed separately to characterize the spectral profile of exposure throughout the day.

For analytical purposes, data were aggregated into time windows across the 24 h cycle (morning, afternoon, and evening), enabling the assessment of temporal exposure patterns and within-subject comparisons between weekdays and weekends [28].

Spectral bands were analyzed based on band-specific integrated irradiance (mW/m^2^), used as a relative indicator of the spectral composition of light exposure [29]. It is important to note that the device does not provide a continuous spectrum nor allow direct conversion of measurements into standardized photometric or α-opic metrics, such as those defined by the CIE S 026/E:2018 (e.g., absolute α-opic irradiances or photopic illuminance in lux) [7]. Nevertheless, data interpretation was informed by its conceptual principles, particularly the functional relevance of short-wavelength lighting as the main spectral component associated with the activation of intrinsically photosensitive retinal ganglion cells (ipRGCs), which are involved in circadian regulation and non-visual light responses [3]. Accordingly, spectral analyses were interpreted in relative terms, as indicators of the functional profile of daily light exposure rather than as absolute measures of α-opic stimulation.

### 4.5. Statistical Analyses

The effects of day type (weekdays vs. weekends) and time of day on light exposure were evaluated using a two-factor repeated-measures analysis of variance (ANOVA), with both factors treated as within-subject variables. Given the rhythmic nature of chronobiological variables, polynomial contrasts were specified for the time factor to identify linear and nonlinear trends across the 24 h cycle.

Time of day was represented by 12 temporal points distributed across the 24 h period. Two-hour intervals were adopted to allow adequate reconstruction of the circadian curve over a 24 h period (n = 12 points), as recommended in chronobiology studies that employ rhythmic modeling, ensuring a balance between temporal resolution and statistical stability [30]. Data normality was assessed and confirmed, supporting the use of parametric analyses. Initially, descriptive statistics (mean, standard deviation, minimum, and maximum) were computed for each time interval, stratified by day-type. Subsequently, repeated-measures ANOVA was conducted to test: (a) the main effect of time, (b) the main effect of day-type, and (c) the time × day-type interaction, assessing whether temporal patterns of light exposure differed between weekdays and weekends.

The assumption of sphericity was evaluated using Mauchly’s test, and when violated, degrees of freedom were corrected using the Greenhouse–Geisser adjustment. When significant main effects or interactions were observed, post hoc analyses were conducted to explore specific differences between day types, with Bonferroni correction applied to control for Type I error. Effect sizes were estimated using partial eta squared (η^2^p), allowing interpretation of the magnitude of observed effects. All analyses were performed using SPSS software (version 24.0).

## 5. Conclusions

In adolescents enrolled in full-time schooling regimes, the temporal organization of light exposure constitutes a key element at the interface between biological time and social time. The findings of the present study indicate that systematic differences between weekdays and weekends emerge predominantly in the shape and amplitude of diurnal and early evening light exposure profiles, rather than as global shifts in the sleep–wake rhythm. This pattern suggests that social time operates as a continuous environmental modulator, capable of altering the everyday light context without necessarily compromising basal circadian stability.

## Figures and Tables

**Table 1:** Comparison between weekdays (DS) and weekends (FDS) in sleep parameters.

Variable	Group	Mean (Min)	Standard Error	95% CI	t (df = 30)	p	Cohen’s d
Total time in bed	DS	504.75	11.48	481.70–526.68	2.096	0.045 *	0.74
	FDS	467.57	13.52	442.40–492.79			
Total sleep time	DS	452.00	13.75	425.74–478.95	2.551	0.016 *	0.90
	FDS	398.86	15.66	370.78–428.78			
Sleep latency	DS	3.80	0.48	2.85–4.79	0.956	0.347	0.34
	FDS	3.18	0.43	2.40–4.04			
WASO (min)	DS	52.27	5.05	43.14–62.14	−0.331	0.743	−0.12
	FDS	55.63	8.82	39.83–73.54			

Note: DS = weekdays; FDS = weekends; * *p* < 0.05.

**Table 2:** Parameter estimates of light exposure, expressed as integrated irradiance (mW/m^2^), by time interval and spectral band (DS − FDS).

Intervale (h)	BL B [IC 95%]	p	η2p	GL B [IC 95%]	p	η2p	RL B [IC 95%]	p	η2p
00:00–02:00	−0.066 [−0.103; −0.029]	<0.001	0.005	−0.079 [−0.121; −0.038]	<0.001	0.003	−0.031 [−0.052; −0.010]	<0.01	0.002
02:00–04:00	−0.003 [−0.004; −0.002]	<0.001	0.005	−0.007 [−0.009; −0.004]	<0.001	0.006	−0.003 [−0.004; −0.002]	<0.001	0.005
04:00–06:00	+0.035 [−0.030; 0.101]	ns	0.001	+0.089 [0.020; 0.158]	0.012	0.001	+0.041 [0.010; 0.072]	<0.01	0.001
06:00–08:00	+2.334 [−0.980; 5.649]	ns	0.001	+3.608 [−1.039; 8.256]	0.128	0.001	+1.927 [−1.058; 4.912]	ns	0.000
08:00–10:00	−85.890 [−120.2; −51.6]	<0.001	0.006	−122.542 [−157.124; −87.961]	<0.001	0.009	−80,317 [−107,0; −53,6]	<0.001	0.006
10:00–12:00	−81.140 [−110.4; −51.9]	<0.001	0.012	−137.618 [−162.801; −112.435]	<0.001	0.021	−75.127 [−92.5; −57.7]	<0.001	0.013
12:00–14:00	−35.155 [−58.9; −11.4]	<0.001	0.002	−61.388 [−85.374; −37.402]	<0.001	0.005	−36.445 [−53.3; −19.6]	<0.001	0.003
14:00–16:00	−51.757 [−77.3; −26.2]	<0.001	0.004	−58.656 [−87.425; −29.887]	<0.001	0.003	−32,315 [−54,0; −10,6]	<0.01	0.002
16:00–18:00	−11.799 [−19.8; −3.8]	<0.01	0.002	−16.252 [−25.973; −6.530]	0.001	0.002	−9.628 [−16.1; −3.1]	<0.01	0.002
18:00–20:00	+0.438 [0.212; 0.664]	<0.001	0.007	+0.778 [0.537; 1.019]	<0.001	0.007	+0.417 [0.293; 0.541]	<0.001	0.008
20:00–22:00	+0.565 [0.331; 0.799]	<0.001	0.14	+1.181 [0.955; 1.407]	<0.001	0.019	+0.688 [0.564; 0.811]	<0.001	0.022
22:00–24:00	−0.597 [−0.811; −0.383]	<0.001	0.16	−1.075 [−1.303; −0.847]	<0.001	0.015	−0.487 [−0.597; −0.378]	<0.001	0.014

Note: B represents the estimated difference between weekdays (DS) and weekends (FDS) (DS − FDS). Negative values indicate greater exposure on weekends, whereas positive values indicate greater exposure on weekdays. CI = 95% confidence interval. ns = not significant (*p* ≥ 0.05). Partial eta squared (η^2^p) corresponds to the effect size and will be reported for all spectral bands after consolidation of the estimates. RL = red light; GL = green light.

## Data Availability

The original contributions presented in this study are included in the article. Further inquiries can be directed to the corresponding author.
